# The risk of chronic obstructive pulmonary disease in patients with rheumatoid arthritis: a real-world cohort study from 136,821 patients

**DOI:** 10.3389/fimmu.2025.1624223

**Published:** 2025-07-08

**Authors:** Tianlun Kang, Yajing Xi, Xiaoping Liu, Tangliang Qian, Siyi Lu, Leqing Li, Zhi Liu, James Cheng-Chung Wei, Xiujuan Hou

**Affiliations:** ^1^ Department of Rheumatism, Dongfang Hospital, Beijing University of Chinese Medicine, Beijing, China; ^2^ Department of Traditional Chinese Medicine, Fu Xing Hospital, Capital Medical University, Beijing, China; ^3^ Emergency Department, Dongfang Hospital, Beijing University of Chinese Medicine, Beijing, China; ^4^ Department of Allergy, Immunology & Rheumatology, Chung Shan Medical University Hospital, Taichung, Taiwan; ^5^ Institute of Medicine, Chung Shan Medical University, Taichung, Taiwan; ^6^ Graduate Institute of Integrated Medicine, China Medical University, Taichung, Taiwan; ^7^ Office of Research and Development, Asia University, Taichung, Taiwan; ^8^ Department of Nursing, Chung Shan Medical University, Taichung, Taiwan

**Keywords:** rheumatoid arthritis, chronic obstructive pulmonary disease, comparative cohort study, TriNetX, propensity score matching, epidemiology

## Abstract

**Objective:**

To compare the risk of chronic obstructive pulmonary disease (COPD) between patients newly diagnosed with RA and individuals without RA. This large-scale study aims to provide novel insights into the association between RA and COPD by evaluating the impact of clinical factors and medications on COPD risk, using robust propensity-score matching.

**Methods:**

This retrospective comparative cohort study utilized data from the TriNetX Research Network, collected on October 9, 2023. Patients newly diagnosed with RA from 2010 to 2021 were compared to non-RA individuals matched on demographics and medical history. Propensity-score matching balanced baseline covariates. The primary outcome was the 5-year risk of newly onset COPD (ICD-10: J41-J44), analyzed using Kaplan-Meier and Cox’s proportional hazards models.

**Results:**

The study included 136,820 pairs of RA and non-RA patients. The 5-year cumulative probability of COPD was 7.36% in the RA cohort versus 5.97% in the non-RA cohort, with a hazard ratio of 1.228 (95% CI = 1.186-1.272). Subgroup analyses showed higher COPD risk in RA patients across different demographics and clinical factors. Males, older patients, higher rheumatoid factor, and higher erythrocyte sedimentation rate increased COPD risk, while DMARDs and systemic NSAIDs reduced it.

**Conclusion:**

RA patients have a significantly higher risk of developing COPD compared to non-RA individuals. These findings underscore the importance of targeted COPD prevention and management in RA patients.

## Introduction

Rheumatoid arthritis (RA) is a chronic inflammatory disorder primarily affecting the joints, but due to its systemic nature, it can also impact other organs, including the lungs (1–3). After the cardiovascular system, the lungs are frequently involved in extra-articular manifestations (EAMs) of RA, ranging from the pleura to blood vessels (4). Chronic obstructive pulmonary disease (COPD) is a progressive lung disease characterized by persistent respiratory symptoms and airflow limitation (5). Historically, RA and COPD were considered distinct entities with separate pathophysiologic mechanisms; however, emerging translational and epidemiologic evidence suggests a complex interplay between the two (6, 7). For example, although smoking is associated with both RA and COPD, multiple analyses have found that the risk of COPD remains significant in RA patients even after adjusting for smoking history (8). However, comprehensive large-scale studies examining the risk of COPD in RA patients compared to the general population are scarce. Although Chiwook Chung and others found that RA is associated with an increased risk of developing COPD in the Korean population (9), further validation is needed with a larger sample database. Additionally, the impact of RA treatments, particularly disease-modifying antirheumatic drugs (DMARDs) and corticosteroids, on the risk of developing COPD remains unclear. Currently, more data is needed to support the true incidence and relative risk of developing COPD after being diagnosed with RA.

This study aims to fill this gap by leveraging data from the TriNetX Research Network, which aggregates de-identified electronic health records (EHR) from multiple healthcare organizations across the United States. This comprehensive dataset allows for robust propensity-score matching and detailed subgroup analyses, providing a clearer picture of the relationship between RA and COPD. By comparing the incidence of COPD in newly diagnosed RA patients with that in non-RA individuals over a 5-year period, this research seeks to provide robust evidence on the heightened risk and inform strategies for better management of COPD in RA patients.

Understanding the interplay between RA and COPD is crucial for developing effective prevention and management strategies. Given the potential for significant morbidity and reduced quality of life associated with COPD in RA patients, identifying those at highest risk and tailoring interventions accordingly could lead to improved outcomes and reduced healthcare burden. Unlike previous studies, our research leverages a comprehensive real-world dataset to not only confirm the RA-COPD link but also identify modifiable risk factors (e.g., DMARDs) and high-risk subgroups, offering actionable insights for clinical practice.

## Methods

### Study design

This was a retrospective comparative cohort study. This design was informed by the previous literature. The risk of COPD was compared between patients newly diagnosed with RA and the individuals without RA.

### Data source

The dataset utilized in this study was collected on October 9, 2023, from the TriNetX Research Network. TriNetX is a federated health research network that aggregates longitudinal electronic health records from 68 healthcare organizations. TriNetX, LLC adheres to the Health Insurance Portability and Accountability Act (HIPAA), a U.S. federal law designed to protect the privacy and security of healthcare data, along with other relevant data privacy regulations applicable to the contributing healthcare organizations. TriNetX is ISO 27001:2013 certified and maintains an Information Security Management System (ISMS) to ensure the protection of healthcare data and compliance with the HIPAA Security Rule. All data displayed on the TriNetX Platform in aggregate form or included in any patient-level data set generated by the platform is de-identified according to the de-identification standards outlined in Section §164.514(a) of the HIPAA Privacy Rule. The de-identification process is verified through a formal determination by a qualified expert as defined in Section §164.514(b)(1) of the HIPAA Privacy Rule. Consequently, as this study involved only de-identified patient records and did not include the collection, use, or transmission of individually identifiable data, it was exempt from Institutional Review Board (IRB) approval. Additionally, this study was conducted in accordance with the principles outlined in the Declaration of Helsinki. The use of TriNetX in this research was approved by the Institutional Review Board of Chung Shan Medical University Hospital (CSMUH), under the approval number CS2-21176.

TriNetX is a platform that de-identifies and aggregates electronic health record (EHR) data, most of which are large academic medical institutions with both inpatient and outpatient facilities at multiple locations, across 50 states in the US. TriNetX Analytics provides web-based and secure access to patient EHR data from hospitals, primary care, and specialty treatment providers, accessible data from the platform include demographics, diagnoses, medications, laboratory values, and procedures. The contributing EHR systems used United Medical Language System biomedical ontologies for coding. TriNetX maps the data to a standard and controlled set of clinical terminologies, for example, mapping disease terms from SNOMED-CT to ICD-10, drug terms from NDCs to RxNorm. The data is then transformed into a proprietary data schema. This transformation process includes an extensive data quality assessment that includes data cleaning (10).

To ensure sufficient data coverage for baseline covariates, we required that all included patients had at least one recorded encounter within 6 months prior to their index date. Patients without records in this look-back window were excluded. Additionally, most TriNetX sites have longitudinal EHR data dating back to January 1, 2000, which ensured that patients diagnosed between 2010 and 2021 had an equal opportunity to contribute covariate data for the baseline period.

### Study population selection

**Figure 1:**
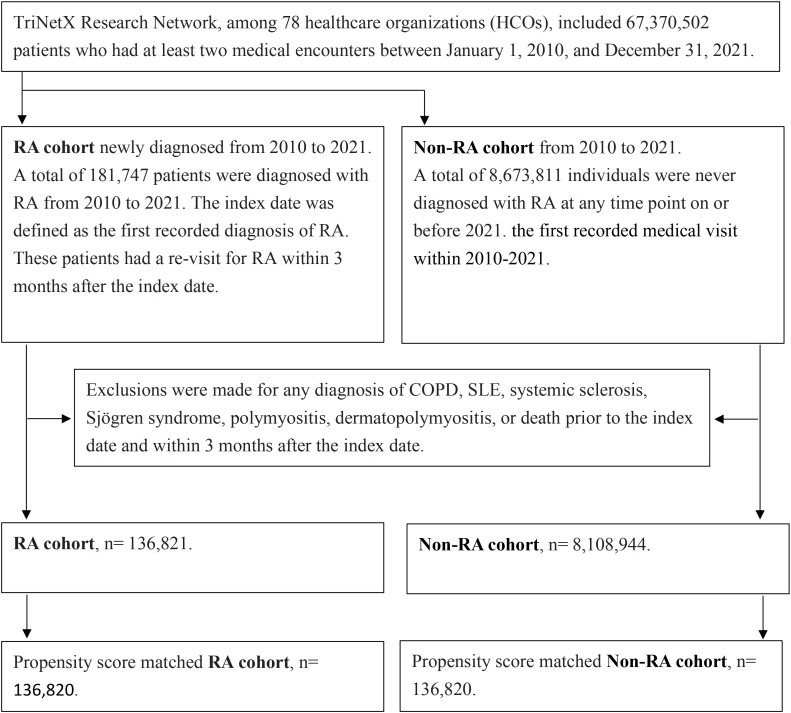
Study flowchart for sample selection. The diagram outlines the identification of the RA and non-RA populations from the TriNetX Research Network, the application of inclusion and exclusion criteria, and the final construction of the matched cohorts. Propensity score matching was based on demographics (age, sex, race, and socioeconomic status), medical utilization (inpatient encounters and emergency visits), smoking status, and comorbidities.

Patients with RA and non-RA were identified from the TriNetX network between 2010 and 2021. Exclusion criteria and the steps of cohort refinement and propensity score matching are shown in the diagram (**Figure 1**).

### RA patients newly diagnosed from 2010 to 2021

We identified patients diagnosed with rheumatoid arthritis (ICD-10: M05-M05.9, M06.0, M06.8, and M06.9) (11) from 2010 to 2021, excluding prevalent RA cases with visits prior to 2010 to ensure incident cases only. The index date was defined as the first recorded RA diagnosis. To establish a clean cohort for assessing COPD risk, we excluded patients with: (1) pre-existing COPD to focus on incident cases; (2) other systemic autoimmune diseases (SLE, systemic sclerosis, Sjögren syndrome, polymyositis, or dermatopolymyositis) due to their overlapping pulmonary manifestations with RA that could confound results; and (3) deaths within 3 months post-index date to avoid immortal time bias. After these exclusions, we selected 136,821 RA patients with confirmed disease activity (evidenced by an RA revisit within 3 months post-diagnosis) for analysis (**Figure 1**).

### Non-RA patients

Individuals who were never diagnosed with RA at any time on or before 2021 were included. These patients had medical visits between 2010 and 2021. The index date for the non-RA cohort was defined as the first recorded medical visit within this period. After excluding any diagnosis of COPD, SLE, systemic sclerosis, Sjögren syndrome, polymyositis, dermatopolymyositis, or death prior to the index date and within 3 months after the index date, we selected 8,108,944 non-RA patients who had any medical encounter within 3 months after the index date (**Figure 1**).

### Study outcomes and follow-up

The study investigated the 5-year risk of newly onset COPD (ICD-10: J41-J44) among RA patients and non-RA patients. For each participant, follow-up began at the index date and ended at the earlier of the occurrence of the study outcomes or censoring at the last recorded fact within the time window in the patient’s record.

To reduce potential misclassification of non-RA individuals due to incomplete electronic health record (EHR) data, we implemented several design strategies. First, we required that all patients—both RA and non-RA—had at least one medical encounter within 3 months after the index date, ensuring that all included individuals were actively engaged in care during the cohort entry period. Second, patients who had any diagnosis of RA, COPD, systemic lupus erythematosus, systemic sclerosis, Sjögren syndrome, polymyositis, or dermatomyositis either prior to or within 3 months after the index date were excluded to improve the specificity of cohort assignment. Lastly, for all patients, follow-up was censored at the last recorded clinical encounter to mitigate misclassification bias due to loss to follow-up or incomplete data.

### Study co-variates

To account for potential confounders, our study included covariates assessed on the index date or within 6 months prior to the index date. These covariates included demographic details (age, sex, race, and socioeconomic status), baseline medical utilization (including inpatient encounters and emergency visits), and lifestyle factors such as nicotine dependence (ICD-10: F17) as an indicator of smoking habits. Additionally, we considered comorbidities including hypertensive diseases, dyslipoproteinemia, diabetes mellitus, neoplasms, overweight or obesity, sleep disorders, ischemic heart diseases, chronic kidney disease, and cerebrovascular diseases. Furthermore, baseline medications were considered, including systemic corticosteroids, systemic nonsteroidal anti-inflammatory drugs (NSAIDs), topical corticosteroids, topical products for joint and muscular pain, antipruritics, psycholeptics, psychoanaleptics, systemic antibacterials, systemic antimycotics, beta-blocking agents, calcium channel blockers, diuretics, and RA-related medications. Laboratory results such as rheumatoid factor (RF), anti-cyclic citrullinated peptide antibody (Anti-CCP), C-reactive protein (CRP), and erythrocyte sedimentation rate (ESR) were also included in the analysis.

### Analysis plan and statistical analysis

Patient cohorts were propensity-score matched for baseline covariates, including demographics, medical utilization (inpatient encounters and emergency visits), lifestyle factors, and comorbidities. Propensity scores were estimated using multivariable logistic regression, with the dependent variable indicating RA status. Matching was performed using 1:1 greedy nearest-neighbor matching without replacement, applying a caliper width of 0.1 standard deviations of the logit of the propensity score to ensure similarity between matched individuals.

To minimize potential overadjustment, co-medications and laboratory values were excluded from the matching process, but were included in downstream multivariable analyses. The matching algorithm was executed on a centrally pooled covariate matrix aggregated from all participating healthcare organizations in the TriNetX network. To reduce potential bias introduced by record ordering in the matrix, rows were randomized before matching. After matching, 136,820 pairs of RA and non-RA patients were selected for comparative analysis (**Figure 1**).

Time-to-event analyses were conducted using Cox proportional hazards regression to estimate hazard ratios (HRs) and 95% confidence intervals (CIs) for the development of COPD. The proportional hazards assumption was tested using the generalized Schoenfeld residuals approach. Kaplan–Meier estimators were used to visualize cumulative incidence of COPD, with censoring applied at each patient’s last recorded clinical encounter.

Laboratory covariates with missing data were handled using complete case analysis (listwise deletion); no imputation was performed. All statistical tests were two-sided, with statistical significance set at p < 0.05. Analyses were conducted on the TriNetX Analytics platform, which applies standard statistical routines including R’s Survival package (v3.2-3) (12).

We also conducted subgroup analyses to evaluate heterogeneity of COPD risk across strata defined by sex, age, and race. Among RA patients, additional stratified analyses were performed by rheumatoid factor level (25–49.9, 50–99.9, ≥100 vs. <25), anti-CCP level (20–39.9, 40–59.9, ≥60 vs. <20), C-reactive protein (CRP) level (1–14.9, ≥15 vs. <1), erythrocyte sedimentation rate (ESR) level (>20 vs. ≤20), and baseline use (yes vs. no) of DMARDs, systemic corticosteroids, and NSAIDs.

### TriNetX platform-specific analytical modules and workflow

To ensure the reproducibility and transparency of the study, we provide a detailed description of the specific analytical modules and workflow used within the TriNetX Analytics Platform

### Baseline characteristics 

Baseline demographic and clinical characteristics of the propensity-score-matched (PSM) RA and non-RA cohorts were compared using the “Compare Characteristics” module within the TriNetX platform (**Table 1**). This function applies standardized statistical routines to generate descriptive summaries and event estimates. Cumulative incidence of COPD at 1, 3, and 5 years was calculated using Kaplan-Meier survival analysis, and hazard ratios (HRs) with 95% confidence intervals were derived using Cox proportional hazards models. The models were adjusted for propensity score matching and stratified by key demographic subgroups (e.g., sex, age, race). The number of events and total sample size are presented in **Table 1**.

**Table 1:** The cumulative probability and hazard ratio for risk of COPD after index date among propensity score matched RA cohort and non-RA cohort.

Characteristics	n	COPD event	Cumulative probability (95% CI) of COPD			Hazard ratio (95% CI)
			1-year	3-year	5-year	
All PSM cohorts						
RA	136820	6761	1.60% (1.53%-1.67%)	4.61% (4.49%-4.74%)	7.36% (7.18%-7.53%)	1.228 (1.186-1.272)
Non-RA	136820	5832	1.29% (1.22%-1.35%)	3.90% (3.79%-4.02%)	5.97% (5.82%-6.12%)	Reference
Female						
RA	101051	4556	1.42% (1.35%-1.50%)	4.21% (4.07%-4.35%)	6.69% (6.49%-6.88%)	1.258 (1.205-1.313)
Non-RA	101051	3852	1.20% (1.13%-1.27%)	3.44% (3.31%-3.56%)	5.30% (5.14%-5.47%)	Reference
Male						
RA	33810	2042	2.12% (1.96%-2.29%)	5.72% (5.44%-6.01%)	9.17% (8.78%-9.58%)	1.300 (1.219-1.388)
Non-RA	33810	1648	1.56% (1.42%-1.70%)	4.59% (4.34%-4.85%)	7.06% (6.72%-7.41%)	Reference
Age <60						
RA	45634	1702	1.03% (0.96%-1.12%)	3.06% (2.92%-3.21%)	4.96% (4.76%-5.16%)	1.560 (1.464-1.662)
Non-RA	45634	1266	0.68% (0.61%-0.74%)	2.02% (1.91%-2.14%)	3.20% (3.04%-3.36%)	Reference
Age ≥60						
RA	87355	4869	1.61% (1.54%-1.69%)	4.67% (4.54%-4.80%)	7.44% (7.26%-7.63%)	1.302 (1.256-1.350)
Non-RA	87355	4403	1.24% (1.18%-1.31%)	3.67% (3.56%-3.79%)	5.72% (5.57%-5.88%)	Reference
White						
RA	87811	4695	1.74% (1.65%-1.83%)	4.88% (4.72%-5.04%)	7.67% (7.46%-7.90%)	1.313 (1.258-1.370)
Non-RA	87811	3849	1.30% (1.23%-1.38%)	3.84% (3.70%-3.98%)	5.84% (5.66%-6.03%)	Reference
Non-White						
RA	22574	1077	1.53% (1.37%-1.71%)	4.44% (4.14%-4.76%)	7.04% (6.63%-7.48%)	1.132 (1.039-1.233)

### Cumulative incidence and hazard ratios

**Figure 2:**
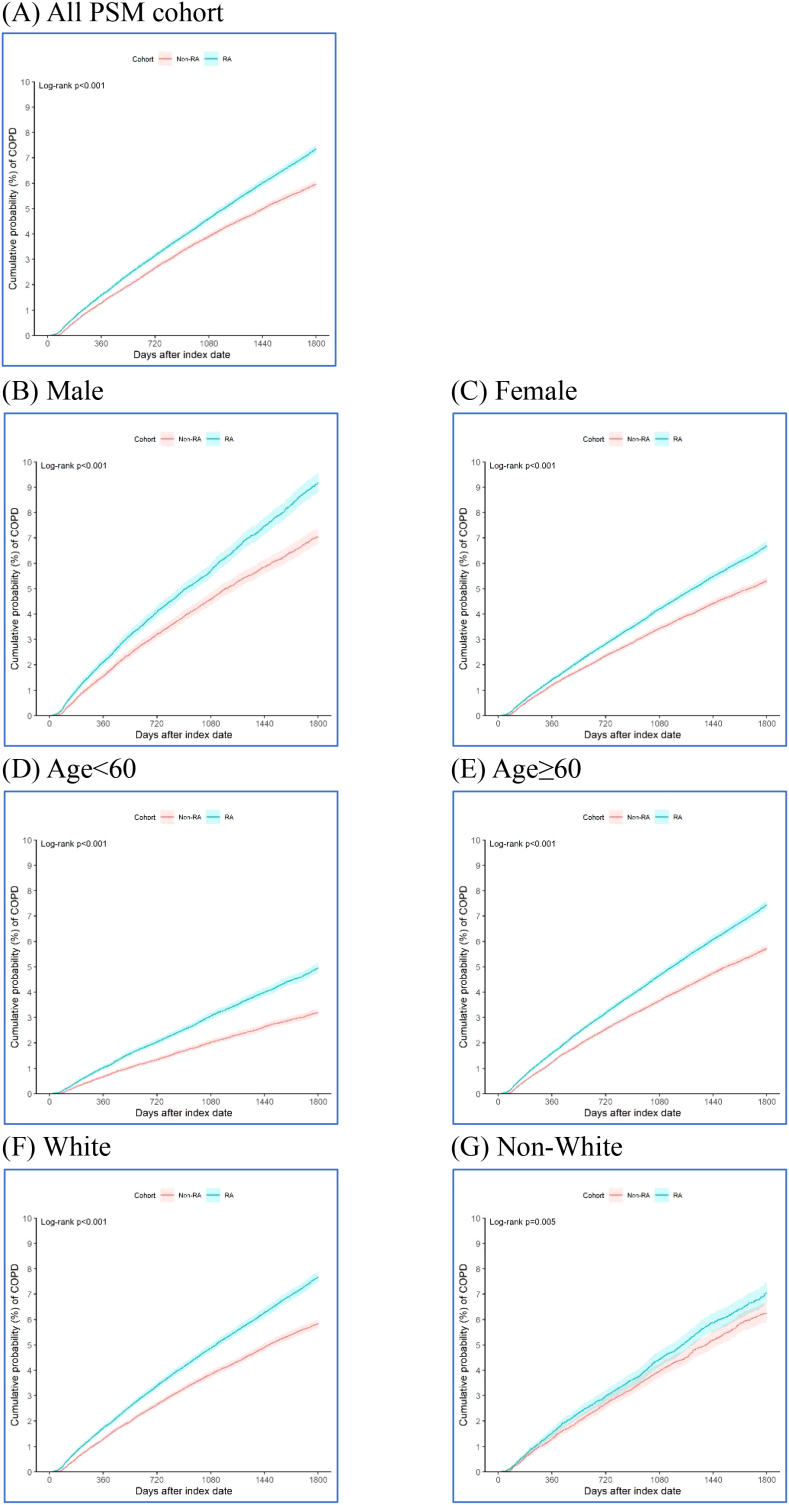
Kaplan-Meier curves showing the cumulative incidence of COPD in RA and non-RA cohorts, stratified by sex, age, and race **(A-G)**.

**Figure 3:**
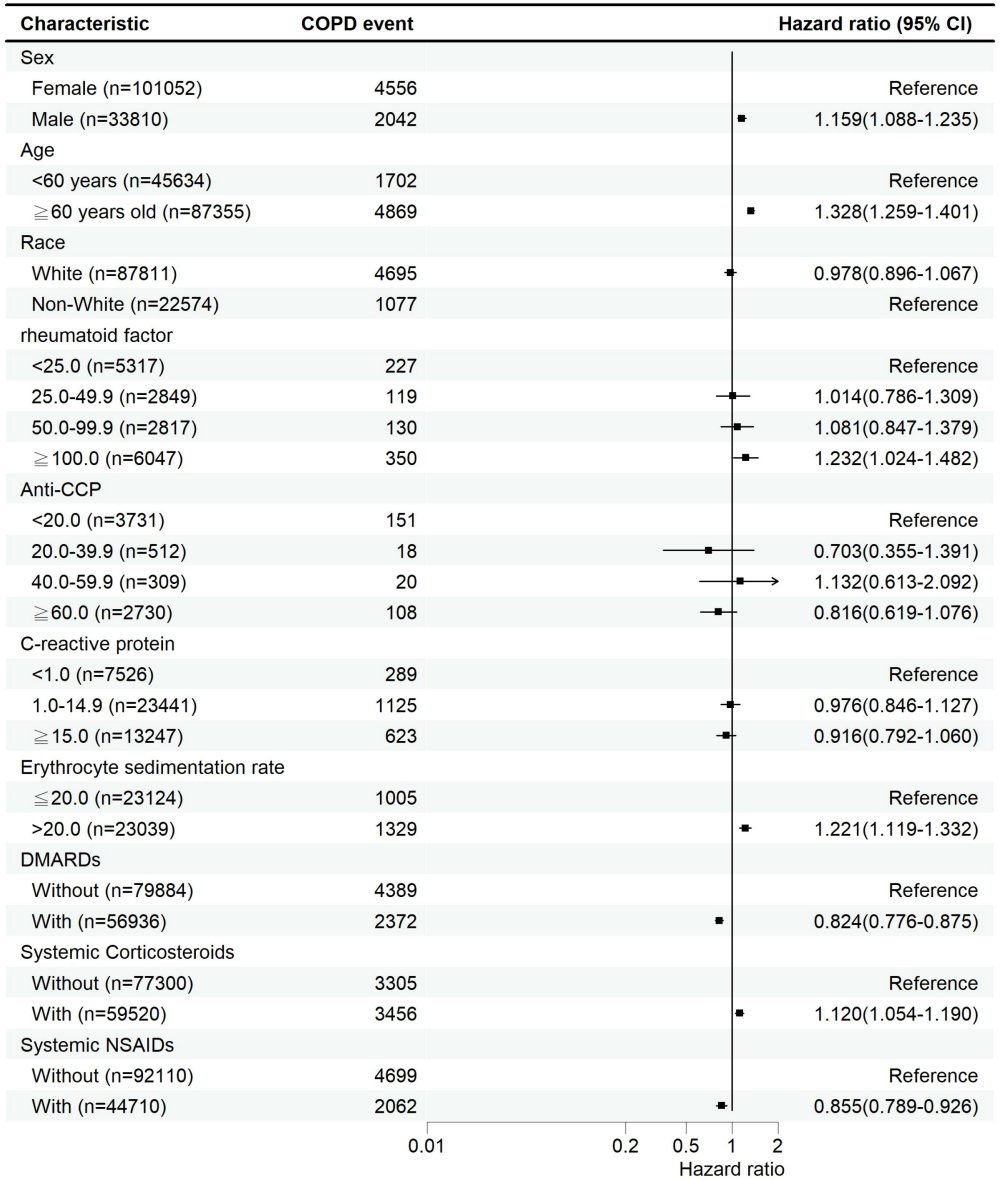
Adjusted hazard ratios for COPD risk factors among RA patients from multivariable Cox proportional hazards models, stratified by demographic and clinical characteristics. Missing laboratory values were handled by complete case analysis.

To assess the incidence of COPD and estimate hazard ratios between matched cohorts, the “Compare Outcomes” module was applied. The workflow was as follows: “Compare Outcomes”→”How do outcomes compare between cohorts?”→”Index Event.”Kaplan–Meier survival analysis and Cox proportional hazards modeling were performed. The hazard ratios and 95% confidence intervals were calculated using the survival package (v3.2-3) embedded in the TriNetX Analytics Platform (**Figures 2**, **3**).

The results presented in **Figure 3** were derived from a single multivariable Cox proportional hazards model. This model included adjustment for demographics (age, sex, race, and socioeconomic status), baseline medical utilization (inpatient and emergency visits), smoking status, comorbidities, co-medications, and laboratory values. Patients with missing laboratory data were excluded via complete case analysis; no imputation was performed.

### Longitudinal laboratory data (1-year, 3-year, and 5-year follow-up)

To obtain laboratory values at specified time points after the index event, we used the “Advanced Explore Cohort” module. The workflow was: “Index Event”→”Explore Characteristics”→”Explore Outcomes,” with appropriate time filters applied to extract laboratory data at 1, 3, and 5 years post-RA diagnosis. Only patients with complete laboratory results at each time point were included in the descriptive analysis.

All data extractions and statistical analyses were conducted exclusively within the TriNetX Analytics Platform between October 9 and October 20, 2023. Propensity score matching, censoring, and statistical testing were automatically executed by the platform based on the defined cohorts and time windows. In accordance with the TriNetX data use policy, no patient-level data were downloaded or exported during this study.

## Results

### Characteristics of study cohorts

This study initially identified 181,747 patients diagnosed with RA and 8,673,811 individuals never diagnosed with RA from 2010 to 2021. After excluding ineligible participants and performing propensity score matching, there were 136,820 pairs of RA and non-RA patients for analysis (**Figure 1**). Before propensity score matching, in the RA cohort, the mean age at the index date was 58.3 ± 15.6 years, and 73.9% were female. Most baseline characteristics were imbalanced between the RA cohort and the non-RA cohort, as defined by a standardized mean difference (SMD) > 0.1. After propensity score matching, the baseline demographics, medical utilization (inpatient encounters and emergency visits), lifestyle factors, and comorbidities were balanced between the RA cohort and the non-RA cohort (**Table 2**).

**Table 2:** Baseline characteristics.

Characteristics	*Before PSM*			*After PSM*		
	RA	Non-RA	SMD	RA	Non-RA	SMD
N	136821	8108944		136820	136820	
Age at index date	58.3 ± 15.6	35.3 ± 25.4	1.0879	58.3 ± 15.6	58.3 ± 15.6	0.0010
Sex						
Female	101052 (73.9%)	4479883 (55.2%)	0.3966	101051 (73.9%)	101054 (73.9%)	0.0000
Male	33810 (24.7%)	3493887 (43.1%)	0.3957	33810 (24.7%)	33785 (24.7%)	0.0004
Race						
White	87811 (64.2%)	4839811 (59.7%)	0.0927	87811 (64.2%)	87857 (64.2%)	0.0007
Black or African American	15815 (11.6%)	1314318 (16.2%)	0.1348	15815 (11.6%)	15827 (11.6%)	0.0003
Asian	6569 (4.8%)	307624 (3.8%)	0.0497	6569 (4.8%)	5748 (4.2%)	0.0289
Smoking	8240 (6.0%)	258321 (3.2%)	0.1357	8240 (6.0%)	5804 (4.2%)	0.0808
Socioeconomic and psychosocial circumstances	965 (0.7%)	106665 (1.3%)	0.0610	965 (0.7%)	1175 (0.9%)	0.0174
Baseline medical utilization						
Inpatient Encounter	22086 (16.1%)	533092 (6.6%)	0.3050	22085 (16.1%)	22018 (16.1%)	0.0013
Emergency	17133 (12.5%)	720359 (8.9%)	0.1179	17133 (12.5%)	12877 (9.4%)	0.0997
Baseline co-morbidity						
Hypertensive diseases	49413 (36.1%)	1526806 (18.8%)	0.3947	49412 (36.1%)	47822 (35.0%)	0.0243
Disorders of lipoprotein metabolism	32700 (23.9%)	1388362 (17.1%)	0.1685	32700 (23.9%)	32685 (23.9%)	0.0003
Diabetes mellitus	19896 (14.5%)	610158 (7.5%)	0.2254	19896 (14.5%)	19890 (14.5%)	0.0001
Neoplasms	16520 (12.1%)	647365 (8.0%)	0.1365	16520 (12.1%)	16462 (12.0%)	0.0013
Overweight or obesity	13939 (10.2%)	655626 (8.1%)	0.0730	13939 (10.2%)	13979 (10.2%)	0.0010
Sleep disorders	13053 (9.5%)	413445 (5.1%)	0.1712	13052 (9.5%)	12955 (9.5%)	0.0024
Ischemic heart diseases	11399 (8.3%)	253381 (3.1%)	0.2255	11398 (8.3%)	11354 (8.3%)	0.0012
Chronic kidney disease	7348 (5.4%)	165794 (2.0%)	0.1767	7348 (5.4%)	6137 (4.5%)	0.0409
Cerebrovascular diseases	5543 (4.1%)	122524 (1.5%)	0.1550	5542 (4.1%)	4655 (3.4%)	0.0342
Baseline Lab examination						
Rheumatoid Factor						
<25	5317 (3.9%)	9685 (0.1%)	0.2713	5317 (3.9%)	274 (0.2%)	0.2628
25-49	2849 (2.1%)	608 (0.0%)	0.2051	2849 (2.1%)	22 (0.0%)	0.2038
50-99	2817 (2.1%)	273 (0.0%)	0.2045	2817 (2.1%)	12 (0.0%)	0.2037
≥100	6047 (4.4%)	214 (0.0%)	0.3038	6047 (4.4%)	18 (0.0%)	0.3028
Anti-cyclic citrullinated peptide Antibody						
<20	3731 (2.7%)	3528 (0.0%)	0.2311	3731 (2.7%)	126 (0.1%)	0.2249
20-39	512 (0.4%)	124 (0.0%)	0.0862	512 (0.4%)	<11	0.0844
40-59	309 (0.2%)	18 (0.0%)	0.0672	309 (0.2%)	<11	0.0643
≥60	2730 (2.0%)	254 (0.0%)	0.2013	2730 (2.0%)	11 (0.0%)	0.2005
C-reactive protein						
<1	7526 (5.5%)	35522 (0.4%)	0.3016	7526 (5.5%)	919 (0.7%)	0.2820
1-14	23441 (17.1%)	98465 (1.2%)	0.5737	23441 (17.1%)	2856 (2.1%)	0.5280
≥15	13247 (9.7%)	48521 (0.6%)	0.4204	13246 (9.7%)	1809 (1.3%)	0.3729
Erythrocyte sedimentation rate						
≤20	23124 (16.9%)	121466 (1.5%)	0.5529	23123 (16.9%)	2908 (2.1%)	0.5204
>20	23039 (16.8%)	57287 (0.7%)	0.5949	23039 (16.8%)	2121 (1.5%)	0.5487
Baseline medications						
Corticosteroids for systemic use	59520 (43.5%)	936851 (11.6%)	0.7660	59520 (43.5%)	20708 (15.1%)	0.6558
Systemic NSAIDs	44710 (32.7%)	797224 (9.8%)	0.5816	44710 (32.7%)	18150 (13.3%)	0.4743
Topical corticosteroids	41044 (30.0%)	1132843 (14.0%)	0.3945	41044 (30.0%)	23853 (17.4%)	0.2987
Topical products for joint and muscular pain	25579 (18.7%)	589836 (7.3%)	0.3448	25579 (18.7%)	12101 (8.8%)	0.2888
Antipruritics, incl. antihistamines, anesthetics	29358 (21.5%)	595480 (7.3%)	0.4104	29358 (21.5%)	16756 (12.2%)	0.2479
Psycholeptics	31700 (23.2%)	770760 (9.5%)	0.3761	31699 (23.2%)	23035 (16.8%)	0.1588
Psychoanaleptics	25839 (18.9%)	873755 (10.8%)	0.2297	25839 (18.9%)	21361 (15.6%)	0.0867
Antibacterials for systemic use	37486 (27.4%)	1460873 (18.0%)	0.2254	37485 (27.4%)	29997 (21.9%)	0.1272
Antimycotics for systemic use	3891 (2.8%)	180523 (2.2%)	0.0393	3890 (2.8%)	3787 (2.8%)	0.0046
Beta blocking agents	21834 (16.0%)	476396 (5.9%)	0.3276	21833 (16.0%)	17933 (13.1%)	0.0809
Calcium channel blockers	14326 (10.5%)	353537 (4.4%)	0.2348	14325 (10.5%)	12826 (9.4%)	0.0367
Diuretics	20604 (15.1%)	542040 (6.7%)	0.2715	20603 (15.1%)	18675 (13.6%)	0.0402
RA related medications						
Hydroxychloroquine	19074 (13.9%)	4823 (0.1%)	0.5654	19074 (13.9%)	186 (0.1%)	0.5605
Sulfasalazine	6077 (4.4%)	2338 (0.0%)	0.3019	6077 (4.4%)	75 (0.1%)	0.2992
Methotrexate	31715 (23.2%)	8477 (0.1%)	0.7711	31715 (23.2%)	230 (0.2%)	0.7676
Leflunomide	5072 (3.7%)	559 (0.0%)	0.2767	5072 (3.7%)	19 (0.0%)	0.2759
Cyclosporine	1010 (0.7%)	8089 (0.1%)	0.0990	1010 (0.7%)	333 (0.2%)	0.0708
Azathioprine	1001 (0.7%)	4671 (0.1%)	0.1077	1001 (0.7%)	156 (0.1%)	0.0953
Etanercept	3858 (2.8%)	1247 (0.0%)	0.2389	3858 (2.8%)	26 (0.0%)	0.2384
Adalimumab	4612 (3.4%)	4395 (0.1%)	0.2578	4612 (3.4%)	94 (0.1%)	0.2561
Infliximab	1859 (1.4%)	3284 (0.0%)	0.1587	1859 (1.4%)	51 (0.0%)	0.1592
Certolizumab pegol	563 (0.4%)	327 (0.0%)	0.0896	563 (0.4%)	<11	0.0885
Golimumab	551 (0.4%)	173 (0.0%)	0.0892	551 (0.4%)	<11	0.0875
Abatacept	1489 (1.1%)	76 (0.0%)	0.1481	1489 (1.1%)	<11	0.1468
Rituximab	841 (0.6%)	2564 (0.0%)	0.1029	841 (0.6%)	93 (0.1%)	0.0938
Tocilizumab	872 (0.6%)	396 (0.0%)	0.1120	872 (0.6%)	15 (0.0%)	0.1104
Sarilumab	63 (0.0%)	<11	0.0302	63 (0.0%)	<11	0.0304
Anakinra	96 (0.1%)	163 (0.0%)	0.0359	96 (0.1%)	<11	0.0319

Baseline period was defined as the time interval within 6 months before index date.

### Main outcomes

**Table 1** details the risk of COPD within 5 years after the index date in the RA and non-RA cohorts. For the 136,820 pairs of propensity score matching RA and non-RA cohorts, there were 6,761 COPD cases in the RA cohort and 5,832 COPD cases in the non-RA cohort during the 5-year observation period. In the RA cohort, the cumulative probability of COPD was 1.60% (95% CI = 1.53%-1.67%) at 1 year, 4.61% (95% CI = 4.49%-4.74%) at 3 years, and 7.36% (95% CI = 7.18%-7.53%) at 5 years. In the non-RA cohort, the cumulative probability of COPD was 1.29% (95% CI = 1.22%-1.35%) at 1 year, 3.90% (95% CI = 3.79%-4.02%) at 3 years, and 5.97% (95% CI = 5.82%-6.12%) at 5 years. The hazard ratio of COPD was 1.228 (95% CI = 1.186-1.272) in the RA cohort compared with the non-RA cohort. For subgroup analysis, the hazard ratio of COPD in the RA cohort was 1.300 (95% CI = 1.219-1.388) in the male subgroup, 1.258 (95% CI = 1.205-1.313) in the female subgroup, 1.560 (95% CI = 1.464-1.662) in the age <60 years subgroup, 1.302 (95% CI = 1.256-1.350) in the age ≥60 years subgroup, 1.313 (95% CI = 1.258-1.370) in the White race subgroup, and 1.132 (95% CI = 1.039-1.233) in the non-White race subgroup. Furthermore, the Kaplan-Meier method showed that the RA cohort was associated with a significantly higher risk of cumulative incidence of COPD in the full cohort (Log-rank p<0.001; **Figure 2A**), male subgroup (Log-rank p<0.001; **Figure 2B**), female subgroup (Log-rank p<0.001; **Figure 2C**), age <60 years subgroup (Log-rank p<0.001; **Figure 2D**), age ≥60 years subgroup (Log-rank p<0.001; **Figure 2E**), White race subgroup (Log-rank p<0.001; **Figure 2F**), and non-White race subgroup (Log-rank p=0.005; **Figure 2G**).

### The factors associated with increased risk of COPD in RA patients

**Figure 3** shows the hazard ratio of COPD for various COPD-associated factors, including sex, age, race, rheumatoid factor, anti-CCP, C-reactive protein, erythrocyte sedimentation rate, DMARDs, systemic corticosteroids, and systemic NSAIDs. These hazard ratios are adjusted for demographics (age, sex, race, and socioeconomic status), medical utilization (inpatient encounters and emergency visits), smoking status, comorbidities, co-medications, and laboratory examination data. Compared with females, males had a 15.9% (95% CI = 8.8%-23.5%) increased risk of COPD. Compared with RA patients aged <60 years, those aged ≥60 years had a 32.8% (95% CI = 25.9%-40.1%) increased risk of COPD. Compared with a rheumatoid factor <25, a rheumatoid factor ≥100 increased the risk of COPD by 23.2% (95% CI = 2.4%-48.2%). Compared with an erythrocyte sedimentation rate ≤20, an erythrocyte sedimentation rate >20 increased the risk of COPD by 22.1% (95% CI = 11.9%-33.2%). RA patients receiving DMARDs treatment had a decreased COPD risk (HR = 0.824, 95% CI = 0.776-0.875) and systemic NSAIDs treatment (HR = 0.855, 95% CI = 0.789-0.926). However, systemic corticosteroid treatment increased the risk of COPD (HR = 1.120, 95% CI = 1.054-1.190).

## Discussion

Our study advances the current understanding of RA-associated COPD in three key aspects: (1) the largest matched cohort to date, minimizing confounding; (2) first demonstration of DMARDs’ protective effect against COPD in RA; and (3) stratification of risk by demographic and inflammatory markers, enabling targeted prevention. This study demonstrates that patients newly diagnosed with RA have a significantly increased risk of developing COPD compared to individuals without RA. The cumulative probability of developing COPD within 5 years was 7.36% in the RA cohort versus 5.97% in the non-RA cohort (HR = 1.228, 95% CI = 1.186–1.272). These findings are consistent with earlier studies and underscore the high susceptibility of RA patients to COPD across various demographic and clinical subgroups (13, 14). By utilizing the extensive TriNetX dataset and applying propensity score matching to control for known confounders such as smoking, age, and comorbidities, this study strengthens the existing evidence base. However, we acknowledge that our COPD definition relied on ICD-10 codes and did not include spirometry-based confirmation, which may result in potential misclassification and warrants cautious interpretation. Additionally, interstitial lung disease (ILD), a common pulmonary manifestation of RA, may clinically overlap with COPD and shares similar ICD-10 coding patterns. We did not exclude ILD-related diagnoses to avoid misclassification, as coding may be inconsistent and incomplete. This represents a limitation of the study and should be addressed in future research using imaging or lung function data.

Our study was additionally limited by the lack of spirometry data for GOLD staging in the EHR-based cohort. Future prospective studies incorporating standardized pulmonary function tests are needed to assess whether RA preferentially impacts specific COPD phenotypes (e.g., emphysema-dominant vs. airway-dominant subtypes).

The observed association between RA and COPD may be attributed to multiple factors. RA is characterized by systemic inflammation, which can lead to lung damage and subsequent development of COPD (15, 16). Cytokines and other inflammatory mediators involved in the pathogenesis of RA may also play a role in COPD (17–20). IL-1β and TNF-α are pro-inflammatory cytokines primarily secreted by macrophages (21). TNF signaling is multifaceted in the pathogenesis of RA, activating endothelial cells and recruiting pro-inflammatory cells such as synovial fibroblasts and macrophages, which release pro-inflammatory cytokines like IL-6, IL-1β, and TNF-α (22). IL-1β and TNF-α activate synovial cells to secrete various inflammatory mediators, such as prostaglandins and matrix metalloproteinases, further exacerbating inflammation and tissue destruction. These cytokines also activate osteoblasts and osteoclasts, leading to cartilage and bone degradation, ultimately causing joint destruction and deformity (23, 24). They also promote the activation of T cells and B cells, enhancing the production of other inflammatory mediators, forming a positive feedback loop that sustains and exacerbates the inflammatory state (25, 26). IL-1β is a typical innate immune cytokine associated with COPD and plays a critical role in initiating and maintaining airway inflammation (27). Elevated serum IL-1β levels can serve as a biomarker indicating the potential ongoing exacerbation of COPD (28). Furthermore, TNF-α polymorphisms are associated with clinical features of COPD, including disease progression (29).

Furthermore, the autoimmune nature of RA may lead to pulmonary manifestations beyond typical joint involvement, further increasing the risk of COPD. For instance, ACPA positivity before the onset of RA is significantly associated with an increased risk of COPD, particularly in the pre-RA phase. This may be due to increased citrullination and ACPA production occurring before RA onset, leading to the loss of immune tolerance in the lungs, which can cause chronic airway disease independent of smoking (30). Subgroup analysis in this study revealed that certain demographic and clinical characteristics influence the risk of COPD in RA patients. Men, older age, and patients with higher levels of rheumatoid factor and erythrocyte sedimentation rate were found to have an increased risk. This suggests that more aggressive RA or greater systemic inflammation may enhance susceptibility to COPD. These findings highlight the need for tailored monitoring and management strategies for high-risk RA patients. While CRP is commonly used to assess inflammation, its non-specific nature limits its interpretative power in distinguishing RA-related versus COPD-related inflammation. Future studies should explore the role of autoantibodies such as RF and ACPA in more detail.

Interestingly, the use of DMARDs and systemic NSAIDs was associated with a reduced risk of COPD, while systemic corticosteroids increased the risk. A real-world study of 7,400 patients found that neither conventional nor biological DMARDs raised the risk of acute exacerbations or infections in RA patients with COPD, possibly due to their anti-inflammatory effects (31). Similarly, NSAIDs may reduce COPD risk by mitigating systemic inflammation. In our study, NSAID use was associated with a lower COPD risk (HR = 0.855). However, since we did not exclude asthma patients—and NSAIDs may trigger bronchospasm in this group—this could introduce confounding. Despite this, the protective association remains, suggesting that anti-inflammatory effects may outweigh potential risks in broader populations. Future studies should validate these findings by excluding asthma or performing stratified analyses.

The findings of this study have several clinical implications. Firstly, they highlight the necessity of respiratory monitoring for RA patients, especially those at high risk. Early detection and management of COPD may improve prognosis and quality of life in this population. Secondly, the protective effects of certain RA treatments suggest that optimizing anti-inflammatory therapy for RA may benefit the respiratory system. Clinicians should consider these factors when developing treatment plans for RA patients. The strengths of this study include its large sample size, use of comprehensive and diverse datasets, and rigorous methodology. Of note, methotrexate, a commonly used DMARD, has been associated with pulmonary toxicity, including interstitial lung disease and fibrosis. We recognize this as a potential confounder and plan to perform sensitivity analyses stratifying patients by MTX use (current, past, and cumulative dose) in future studies.

Additional limitations should be noted. First, the retrospective design precludes causal inference. Second, the diagnosis of COPD was based solely on ICD-10 codes without spirometric confirmation, which may reduce diagnostic specificity. Third, the TriNetX dataset is primarily derived from U.S.-based, predominantly White populations, potentially limiting the generalizability of our findings. Fourth, detailed information on nicotine exposure (e.g., pack-years, duration, cessation) was unavailable, which may affect the accuracy of smoking-related adjustments. Fifth, although we excluded patients with malignant neoplasms using SOC codes, future analyses may benefit from restricting to biopsy-confirmed cases to rule out lung metastases.

Finally, we acknowledge a potential source of detection bias: individuals experiencing subclinical or early respiratory symptoms suggestive of COPD may seek medical care more frequently, thereby increasing the likelihood of RA diagnosis due to heightened clinical attention. This could result in an overestimation of the temporal association between RA onset and subsequent COPD development. Future studies incorporating symptom onset timelines and longitudinal clinical records may help disentangle the directionality of this relationship.

While we acknowledge that the COPD diagnosis in our study was based solely on ICD-10 codes without spirometric confirmation, prior validation studies have suggested that ICD-based COPD definitions demonstrate a moderate to high level of specificity (32). Given this, potential misclassification is likely to be non-differential between matched RA and non-RA groups, which would bias the observed association toward the null rather than inflate it.

Additionally, to mitigate this limitation, we applied strict exclusion criteria for baseline COPD and required follow-up medical encounters within 3 months of cohort entry, ensuring continuous engagement with healthcare services. Furthermore, smoking status—a key confounder—was accounted for in propensity score matching, and nicotine dependence was included in multivariable adjustment. These steps reduce the likelihood that diagnostic misclassification substantially alters the observed association.

Lastly, our study is subject to inherent limitations of EHR-based research. The TriNetX platform does not universally link with claims data, limiting the ability to verify continuous healthcare coverage or capture external care. This raises the potential for misclassification, particularly among non-RA patients with incomplete records. To mitigate this, we required all patients to have at least one clinical encounter within 3 months after the index date and censored follow-up at the last available record. While these steps reduce bias, some degree of residual misclassification remains possible.

In addition, defining the non-RA cohort based on the absence of RA diagnosis throughout the entire study period may introduce selection bias and immortal time bias, as these individuals are by definition “RA-free” in the future. This may affect the comparability and generalizability of the control group. Although we mitigated this by matching index dates within the same calendar quarter between RA and non-RA patients, future studies could consider using risk-set sampling based on calendar time to more rigorously control for time-related bias.

## Conclusion

In conclusion, our study highlights the elevated risk of COPD in newly diagnosed RA patients and emphasizes the need for vigilant respiratory monitoring, especially in high-risk groups. Tailoring RA treatment to mitigate systemic inflammation while preserving pulmonary health may improve long-term outcomes in this vulnerable population. Future prospective studies incorporating spirometric data, detailed smoking history, autoantibody profiles, and treatment exposure metrics will further elucidate this important clinical relationship.

## Data Availability

Publicly available datasets were analyzed in this study. This data can be found here: https://trinetx.com/.
